# Feasibility Study and Preliminary Results of Prognostic Value of Bone SPECT-CT Quantitative Indices for the Response Assessment of Bone Metastatic Prostate Carcinoma to Abiraterone

**DOI:** 10.3389/fmed.2019.00342

**Published:** 2020-01-22

**Authors:** Romain de Laroche, David Bourhis, Philippe Robin, Olivier Delcroix, Solène Querellou, Jean-Pierre Malhaire, Friederike Schlurmann, Vincent Bourbonne, Pierre-Yves Salaün, Ulrike Schick, Ronan Abgral

**Affiliations:** ^1^Nuclear Medicine Department, University Hospital Morvan, Brest, France; ^2^Université de Bretagne Occidentale, Brest, France; ^3^EA 3878 GETBO IFR 148, Brest, France; ^4^Radiation Therapy Department, University Hospital Morvan, Brest, France; ^5^Medical Oncology Department, University Hospital Morvan, Brest, France; ^6^LaTIM, INSERM, UMR 1101, University of Brest, ISBAM, UBO, UBL, Brest, France

**Keywords:** prostate cancer, bone scintigraphy, SPECT-CT, quantification, prognosis

## Abstract

**Objective:** We assessed the prognostic value of quantitative indices extracted from bone SPECT-CT to evaluate the response of bone metastatic castrate-resistant prostate cancer (BmCRPC) to abiraterone.

**Methods:** Consecutive patients with BmCRPC initiating treatment with abiraterone from March 2014 to March 2015 were prospectively included. Three 2-bed SPECT-CT [at baseline [M0], after 3 months [M3], and 6 months [M6] of treatment], were planned (Symbia Intevo^®^, Siemens). SPECT data were reconstructed using an Ordered Subset Conjugate Gradient Minimization (OSCGM) algorithm allowing SUV quantification. SUVmax and SUVpeak of the highest uptake lesion were measured in each SPECT-CT. Total Neoplastic Osteoblastic Metabolic Volume (NOMV) was assessed. PSA level was recorded at baseline, M3, and M6 of treatment. Overall survival (OS), progression-free survival (PFS), and disease-specific survival (DSS) were calculated.

**Results:** Nineteen patients aged 71.1 ± 7.7 years were included. Low M0 SUVmax was significantly predictive of longer OS (*p* = 0.04). Low NOMV at M0 were significantly predictive of longer PFS (*p* = 0.02). Patients with increase of at least 12.5% of the SUVpeak of the highest uptake lesion between M0 and M3 (ΔSUVpeakM0M3) had a significantly longer OS (*p* = 0.03). Patients with increase (or decrease lesser than 25%) of ΔSUVpeakM0M3 had a significantly longer DSS (*p* = 0.01). Patients with increase of NOMV of at least 45% between M0 and M6 had a significantly shorter PFS (*p* < 0.001). Variations of NOMV between M0 and M6 were significantly correlated with PSA variations between M0 and M6 (*rs* = 0.73, *p* = 0.02).

**Conclusions:** Quantitative bone SPECT-CT appears to be a promising tool of BmCRPC assessment. Early flare-up phenomenon seems to predict longer OS.

## Introduction

Prostate adenocarcinoma is one of the most common cancers in men (1), and bone remains the most frequent site of distant metastasis. During the last two decades, the prostate cancer specific mortality has greatly decreased thanks to new therapeutic molecules, among which the second generation of hormonotherapy has revolutionized the treatment of metastatic castrate-resistant prostate cancer (mCRPC). Nevertheless, the prognosis of bone mCRPC (BmCRPC) remains poor, with an overall survival (OS) of <2 years (2).

Abiraterone acetate, an inhibitor of androgen synthesis (CYP17A1 inhibitor) has recently been approved in first line treatment of chemonaïve patients with mCRPC (3). However, the response assessment to this new generation hormone therapy and the identification of treatment-refractory patients remains a real challenge (4). In 2007, the Prostate Cancer Working Group (PCWG) published the PCWG 2 criteria guiding the therapeutic response assessment in prostate cancer trials (5). These criteria are based on composite data including clinical signs (such as general condition and bone pain), Prostatic Specific Antigen (PSA) variations, computed tomography (CT) scan [using RECIST 1.1 criteria (6)] and bone scan results.

PCWG 2 recommends for non-cytotoxic agents such as abiraterone to perform bone scan evaluations every 3 months: at baseline (M0), after 3 months (M3), and 6 months (M6) of treatment. Briefly, 2 new lesions or more between M3 and M6 bone scans define progression. Indeed, new isolated lesions at M3 can be a sign of a flare-up phenomenon, which is an early paradoxical worsening bone scan (7). As a consequence, new lesions on M3 bone scan have to be confirmed within a delay of 6 weeks or more by a second scan, which has to show at least 2 new lesions to conclude to a progression. Thus, these bone scan criteria are simplistic, based only on a visual analysis. Moreover, these criteria refer to the bone scan as a planar imaging while it has been shown that Single Photon Emission Computed Tomography (SPECT) improved sensitivity, specificity, positive and negative predictive values in the oncologic context (8). Finally, PCWG2 acknowledges that there are no validated criteria for response on radionuclide bone scan (5).

The Bone Scan Index (BSI) has been proposed (9) as a quantitative tool to reflect bone metastasis burden in bone scan. Nevertheless, this approach suffers from the limitation to be measured from the whole body planar imaging and estimated by a geometric mean method. While baseline BSI revealed a poor correlation with OS, on-treatment changes in BSI appeared to be a convenient marker of treatment response (10). The recent development of Tc99m quantification tools in SPECT-CT, allowing Standardized Uptake Value (SUV) quantification, offers now the opportunity to assess therapeutic response as well as in positron emission tomography (PET) systems (11, 12). With such a background, we hypothesize that volumetric quantitative biomarker extracted from SPECT acquisition could be correlated with patient outcomes. The objective of this study was also to assess the prognostic value of quantitative indices extracted from bone SPECT-CT to evaluate the response of BmCRPC to abiraterone.

## Materials and Methods

### Patients

Consecutive patients with BmCRPC initiating treatment with abiraterone from March 2014 to March 2015 were prospectively included in one center. Treatment initiation with abiraterone was validated by a multidisciplinary staff. This study was approved by our institutional ethics committee (29BRC17.0189). All participants provided written informed consent. Initial clinical data including age, PSA level, Gleason score, and previous treatment were recorded.

### SPECT-CT Imaging

Included patients were referred to the nuclear medicine department of the Brest University Hospital. Three SPECT-CT [at baseline [M0], after 3 months [M3], and 6 months [M6] of treatment] were planned on the same camera (dual head gamma camera Symbia Intevo^®^, Siemens, Erlangen, Germany).

A two-bed SPECT-CT acquisition (covering axial skeleton) was performed 3 h after injection of 9 MBq/kg (500–800 MBq) of Tc-99m 3,3-diphosphono-1,2-propanedicarboxylic acid (DPD, Teceos^®^, IBA Molecular, Gif-sur-Yvette, France) after the standard whole-body scan. SPECT images were acquired according to the following parameters: 10 s per step acquiring 120 projections with 180° rotation for each camera head, on a 128 × 128 pixel matrix. A low-energy high-resolution parallel-hole collimator was used for the acquisition. The energy window was set at +/– 7.5%, centered on the photon energy peak of Tc-99m (140 keV). SPECT data were reconstructed using an Ordered Subset Conjugate Gradient Minimization (OSCGM) algorithm allowing SUV quantification [xSPECT^®^ (13), Siemens], with the following parameters: 8 iterations and 6 subsets with a 256 × 256 matrix (pixel size 1.9 × 1.9 × 1.9 mm), and a 10 mm FWHM Gaussian post-filter. Scatter correction was applied. CT-based attenuation correction was applied to SPECT images.

The CT acquisition was performed immediately after the SPECT acquisition, from the upper cervical spine to the proximal femora. The CT parameters were as follows: pitch 1, tube voltage 130 kV, automatic mAs control (reference mAs 90), slice thickness 3 mm, matrix 512 × 512, field of view 50.2 cm. The CT reconstruction used a hard filter (B80s).

### Image Analysis

SPECT-CT quantification was performed by the same operator. Different approaches were explored.

#### Static Parameters

Spherical VOI were drawn over the highest uptake target lesions (up to 5) to measure SUVmax and SUVpeak (Syngo.via^®^, Siemens). ΔSUVpeak, corresponding to the SUVpeak variation of the lesion with the highest uptake, was assessed (this lesion could be different between two SPECT-CT scans). The sum of the SUVmax of the highest uptake target lesions (up to 5) (ΣSUVmax) was calculated at M0. ΔΣSUVmax, corresponding to the variation of ΣSUVmax of the same lesions between two SPECT-CT, was calculated. ΔSUVpeak and ΔΣSUVmax were calculated between M0 and M3, between M0 and M6, and between M3 and M6 as follows: ΔSUV_MXMY_ = (SUV_MY_–SUV_MX_)/SUV_MY_.

#### Volumetric Parameters

Total Neoplastic Osteoblastic Metabolic Volume (NOMV) was assessed for each bone SPECT-CT (MIM v6.6, MIMSoftware). A cut-off of 12 of SUV was arbitrarily chosen to manually delineate metastasis. Articular and physiological uptakes were manually excluded from the NOMV. CT images were used to locate the uptakes. SUVmean was defined by the mean of the SUV of all the voxels included in the NOMV. By analogy with Total Lesion Burden in PET, Total Lesion Osteoblastic Metabolism (TLOM) was calculated as follows: TLOM = NOMV × SUVmean.

### Response Assessment and Follow-Up

PSA level was recorded at baseline, at M3, and M6 of treatment with abiraterone. PSA progression was defined as a 25% or greater increase and an absolute increase of 2 ng/mL or more from the nadir, which was confirmed by a second value obtained 3 or more weeks later (5, 14).

SPECT therapeutic response using different quantitative indices was arbitrarily assessed according to usual PET evaluation criteria [PERCIST 1.0 (11) and EORCT 99 (12)] between M3 and M6 in order to avoid flare-up phenomenon. Progressive disease was defined by an increase of the ΔSUVpeak by 30% or higher, or at least one new suspicious uptake according to PERCIST 1.0 criteria. Progressive disease was defined by an increase of the ΔΣSUVmax by 25% or higher, or at least one new suspicious uptake according to EORTC 99 criteria. Partial response was defined by a decrease of the ΔSUVpeak by 30% or higher (PERCIST 1.0) or a decrease of the ΔΣSUVmax by 25% or more (EORTC 99). Complete response was defined by the disappearance of all uptakes. Stable disease was defined by non-response and no progressive disease. Results were compared with PCWG 2 response evaluation and serum PSA level changes.

Routine follow-up was done by the same clinician, according to PSA level variation, clinical signs, CT, and bone scan (visual analysis) evaluations. Progression-free survival (PFS), disease-specific survival (DSS) and OS were calculated. PFS was defined as the number of days from treatment initiation with abiraterone until progression of the disease. DSS only considered death from prostate cancer as an endpoint. OS considered all causes of death as an endpoint.

### Statistical Analysis

Spearman's correlation coefficient *r*_*s*_ was used to correlate PSA level and quantitative SPECT parameters (SUVmax, NOMV, and TLOM) at baseline, and their variations between two scans. The receiver-operating-characteristic (ROC) curve was drawn to estimate the most discriminating threshold for each parameter to maximize the sensitivity and specificity in predicting the PFS, the DSS and the OS. By dichotomizing the parameters based on their cut-point, a Kaplan-Meier survival curve was generated and Log-rank test was used to compare PFS, DSS and OS between each of the two groups (15). Significance level of *p*-value was 0.05. Statistical analysis was performed with XLSTAT software v19.5 (Addinsoft, Paris, France).

## Results

### Patients

**Figure 1:**
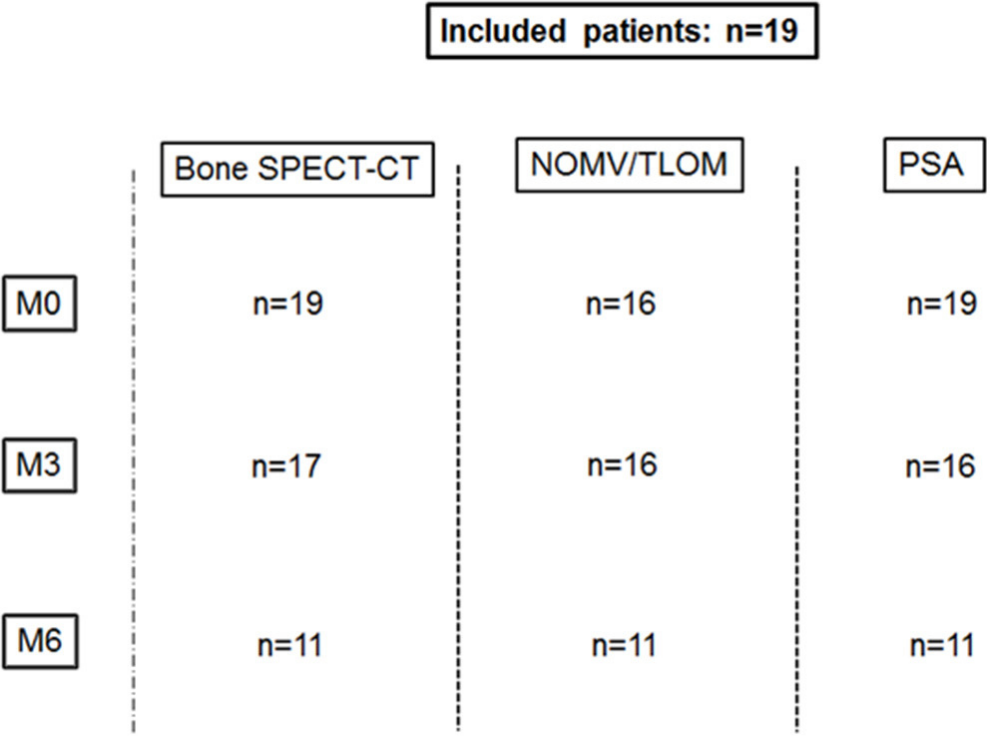
Available data.

Nineteen patients aged 71.1 ± 7.7 years were included from March 2014 to March 2015. Patients' clinical data are provided in Table 1. Because of technical issues, quantification was not available for one of the two bed SPECT acquisitions (thoracic or abdominopelvic) for 3 patients at M0, and for one patient at M3. For one patient, PSA was not available at M3. Two patients died before M3. Six patients stopped abiraterone after M3 because of progression (*n* = 5) or treatment's related side effects (*n* = 1). Available data are summed up in Figure 1. Mean delay between M0 and M3 was 3.4 ± 0.6 months. Mean delay between M0 and M6 was 6.6 ± 0.8 months. Eight patients had extra-bone metastases (lymph nodes *n* = 8, lung *n* = 2, liver = 1).

**Table 1:** Characteristics of the population (*n* = 19).

**Age**	**Mean ± SD**	**74.1 ± 7.7 years**
	Range	59–86 years
Gleason	VI	3
	VII	14
	VIII	1
	IX	1
Previous treatment	Pre-docetaxel	16
	Post-docetaxel	3
Baseline PSA	PSA<20	4
	20<PSA<60	9
	PSA>60	6
Time to castration resistance since diagnosis (yrs)	0–4	7
	5–9	6
	>=10	6

*SD, Standard deviation; PSA, Prostate Specific Antigen*.

Mean follow-up was 2.5 ± 0.5 years (range: 1.7–3.3 years). Ten patients died after a mean follow-up of 0.9 ± 0.6 years. Seven of them died of prostate cancer. The three other patients died from pulmonary embolism, cardiopulmonary arrest, and respiratory distress.

### SPECT Assessment

Median SUVmax ± IQR of the highest uptake lesions (up to 5) in M0, M3, and M6 SPECT images were 37.5 ± 26.7, 37.6 ± 33.6, and 32.4 ± 25.6, respectively. Median SUVpeak ± IQR of the highest uptake lesions (up to 5) at M0, M3, and M6 were, respectively, 32.3 ± 23.6, 33.7 ± 28.5, and 32.3 ± 22.9. Between M3 and M6, therapeutic assessment revealed 1 progressive disease (PD), 9 stable diseases (SD), and 1 partial response (PR) according to PERCIST criteria, 2 PD, 8 SD, and 1 PR according to EORTC 99 criteria, and 1 PD and 10 non PD according to PCWG 2 criteria.

**Figure 2:**
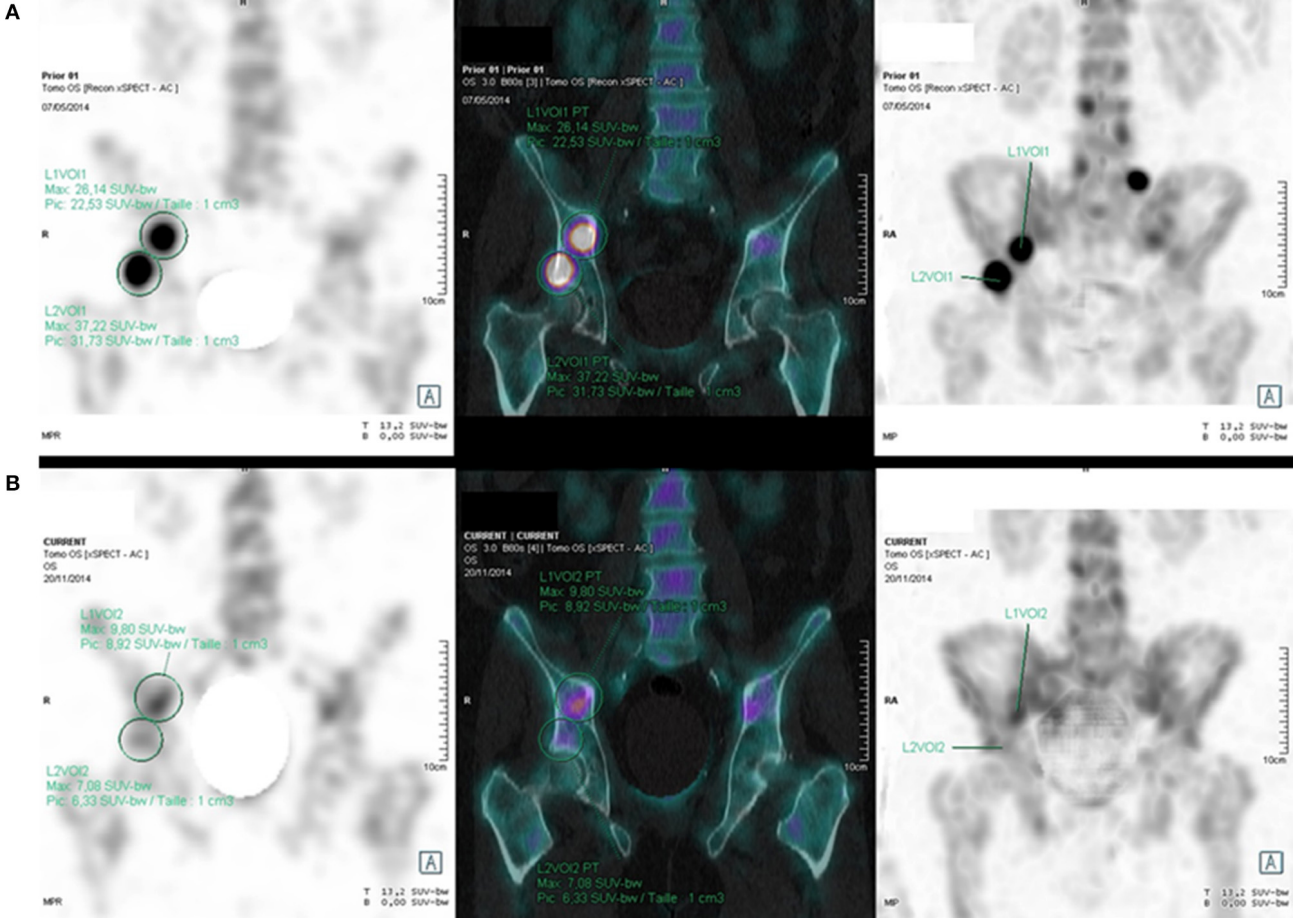
61 years old patient with BmCRPC; SPECT, SPECT-CT, and MIP images show a quantitative regression of the right acetabulum and iliac wing uptakes (SUVmax: respectively, 7.08 vs. 37.22, and 9.80 vs. 26.14) between M0 **(A)** and M6 **(B)** (delay: 6.5 months), with a 97.2% PSA regression (0.72 vs. 26 ng/mL).

Using PERCIST criteria, all the therapeutic responses matched with PCWG 2 evaluation. Using EORTC 99 criteria, 10 responses matched with PCWG 2 evaluation and one mismatched. This patient was classified as progressive according to EORTC 99 criteria, stable according to PERCIST, non-progressive according to PCWG2 criteria, and non-progressive according to PSA evaluation. Figure 2 shows an example of SUV variations between M0 and M6.

**Figure 3:**
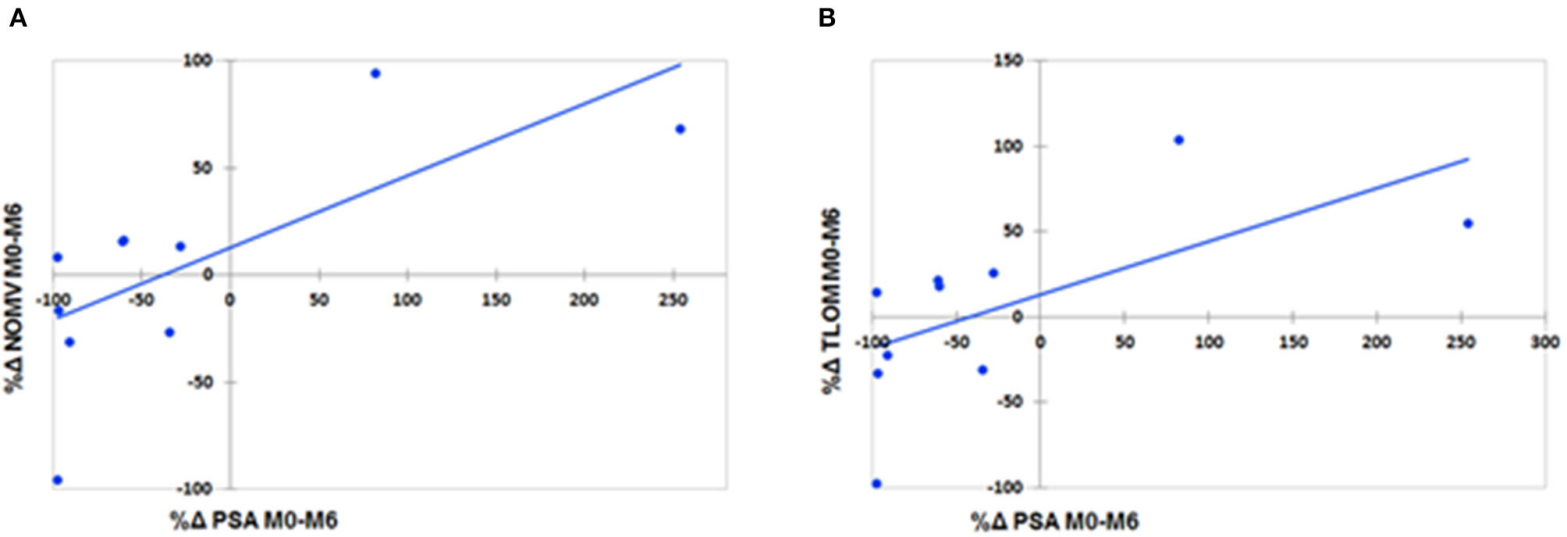
Linear regression graphs of PSA level variations and NOMV variations **(A)** (*r*_s_ = 0.73, *p* = 0.02) and of PSA level variations and TLOM variations **(B)** (*r*_s_ = 0.81, *p* < 0.01) between M0 and M6.

ΔNOMV between M0 and M6 (ΔNOMV_M0M6_) and ΔTLOM between M0 and M6 (ΔTLOM_M0M6_) were significantly associated with ΔPSA between M0 and M6 (ΔPSA_M0M6_) (respectively, *r*_s_ = 0.73, *p* = 0.02 and *r*_s_ = 0.81, *p* < 0.01) (Tables 2, 3). Figure 3 shows the linear regression curves of these two statistically significant associations.

**Table 2:** Correlations between PSA level and SPECT quantitative indices.

	**PSA**_****M0****_	
SUVmax M0	*r*_s_ = 0.12	*p* = 0.61
NOMV M0	*r*_s_ = 0.39	*p* = 0.13
TLOM M0	*r*_s_ = 0.38	*p* = 0.15
	**ΔPSA**_**M0M3**_	
ΔSUVpeak_M0M3_	*r*_s_ = 0.12	*p* = 0.68
ΔΣSUVmax_M0M3_	*r*_s_ = 0.17	*p* = 0.55
ΔNOMV_M0M3_	*r*_s_ = 0.49	*p* = 0.07
ΔTLOM_M0M3_	*r*_s_ = 0.50	*p* = 0.07
	**ΔPSA**_**M0M6**_	
ΔSUVpeak_M0M6_	*r*_s_ = 0.51	*p* = 0.11
ΔΣSUVmax_M0M6_	*r*_s_ = 0.59	*p* = 0.08
ΔNOMV_M0M6_	*r*_s_ = 0.73	*p* = 0.02^*^
ΔTLOM_M0M6_	*r*_s_ = 0.81	*p* < 0.01^*^
	**ΔPSA**_**M3M6**_	
ΔSUVpeak_M3M6_	*r*_s_ = 0.18	*p* = 0.64
ΔΣSUVmax_M3M6_	*r*_s_ = 0.33	*p* = 0.38
ΔNOMV_M3M6_	*r*_s_ = 0.33	*p* = 0.38
ΔTLOM_M3M6_	*r*_s_ = 0.33	*p* = 0.38

* *statistically significant. PSA, Prostate Specific Antigen; SUV, Standardized Uptake Value; NOMV, Neoplastic Osteoblastic Metabolic Volume; TLOM, Total Lesion Osteoblastic Metabolism*.

**Table 3:** Comparison of variations of volumetric SPECT parameters (NOMV and TLOM) with PSA between M0 and M6 (*n* = 10 patients).

**M0–M6**	**PSA increase**	**PSA decrease**
Volumetric SPECT parameters (NOMV and TLOM) increase	2 patients	4 patients
Volumetric SPECT parameters (NOMV and TLOM) decrease	0 patient	4 patients

*PSA, Prostate Specific Antigen; NOMV, Neoplastic Osteoblastic Metabolic Volume; TLOM, Total Lesion Osteoblastic Metabolism*.

### Prognostic Value of Bone SPECT Parameters

**Figure 4:**
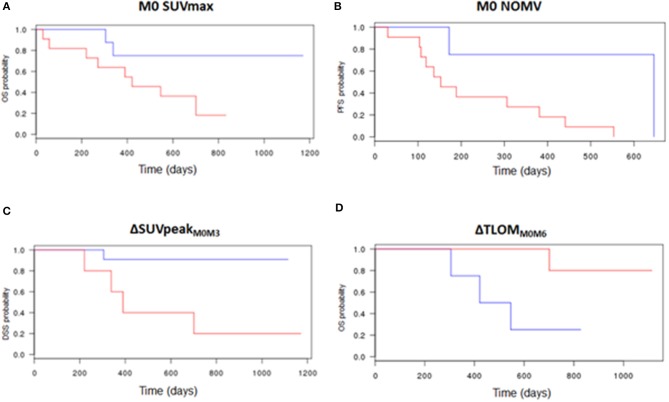
Survival curves according to quantitative bone SPECT parameters. Low M0 SUVmax was predictive of longer OS (cut-off: 52, *p* = 0.04) **(A)**. Low M0 NOMV was predictive of longer PFS (cut-off: 90 mL, *p* = 0.02) **(B)**. The increase or a decrease lesser than 25% of the SUVpeak of the highest uptake lesion between M0 and M3 was predictive of longer DSS (cut-off: −25%, *p* = 0.01) **(C)**. The decrease or an increase lesser than 20% of the TLOM between M0 and M6 was predictive of longer OS (cut-off: +20%, *p* = 0.04) **(D)**. Red curve: part of the population with higher baseline bone SPECT parameters **(A,B)**, or with a variation of their bone SPECT parameters lower than the cut-off **(C,D)**. Blue curve: part of the population with lower baseline bone SPECT parameters **(A,B)**, or with a variation of their bone SPECT parameters higher than the cut-off **(C,D)**.

Table 4 shows the predictive value of scintigraphic parameters regarding OS, DSS, and PFS, and the cut-off used to dichotomize the population into 2 groups according to the ROC curves. Low M0 SUVmax was significantly predictive of longer OS (*p* = 0.04). Low M0 NOMV and TLOM were significantly predictive of longer PFS (respectively, *p* = 0.02 and *p* = 0.04). Patients with increase of at least 12.5% of the SUVpeak of the highest uptake lesion between M0 and M3 (ΔSUVpeak_M0M3_) had a significantly longer OS (*p* = 0.03). Patients with increase (or decrease lesser than 25%) of ΔSUVpeak_M0M3_ had a significantly longer DSS (*p* = 0.01). Patients with increase of at least 4.2% of ΔΣSUVmax between M0 and M3 (ΔΣSUVmax_M0M3_) had a significantly longer DSS (*p* = 0.01). Patients with increase of at least 20% of TLOM between M0 and M6 (ΔTLOM_M0M6_) had a significantly shorter OS (*p* = 0.04). Finally, patients with increase of NOMV of at least 45% or with increase of TLOM of at least 40% between M0 and M6 had a significantly shorter PFS (*p* < 0.001). Figure 4 shows the most significant survival curves of the study.

**Table 4:** Predictive value of quantitative SPECT parameters.

	**OS**	**DSS**	**PFS**
**BASELINE**			
SUVmax M0	*p* = 0.04^*^ (52)	*p* = 0.08 (66)	*p* = 0.30 (63)
M0 NOMV (mL)	*p* = 0.24 (35)	*p* = 0.41 (400)	*p* = 0.02^*^ (90)
M0 TLOM	*p* = 0.24 (600)	*p* = 0.41 (9000)	*p* = 0.04^*^ (1993)
**M0M3**			
ΔSUVpeak_M0M3_ (%)	p = 0.03^*^ (+12.5%)	p = 0.01^*^ (−25%)	*p = 0.22 (+11%)*
ΔΣSUVmax_M0M3_ (%)	*p = 0.12 (+4.2%)*	p = 0.01^*^ (+4.2%)	*p = 0.06 (+14%)*
ΔNOMV_M0M3_ (%)	*p = 0.26 (+2%)*	*p = 0.16 (−2%)*	*p = 0.28 (−2%)*
ΔTLOM_M0M3_ (%)	*p = 0.26 (+10%)*	*p = 0.16 (−10%)*	*p = 0.28 (−10%)*
**M0M6**			
ΔSUVpeak_M0M6_ (%)	*p* = 0.36 (+3%)	*p = 0.06 (−20%)*	*p = 0.36 (−10%)*
ΔΣSUVmax_M0M6_ (%)	*p* = 0.39 (–6%)	*p = 0.20 (+4%)*	*p = 0.09 (+2%)*
ΔNOMV_M0M6_ (%)	*p* = 0.11 (–20%)	*p* = 0.24 (–20%)	*p* < 0.001^*^ (+45%)
ΔTLOM_M0M6_ (%)	*p* = 0.04^*^ (+20%)	*p* = 0.24 (+40%)	*p* < 0.001^*^ (+40%)
**M3M6**			
ΔSUVpeak_M3M6_ (%)	*p* = 0.30 (–15%)	*p = 0.20 (−4.2%)*	*p* = 0.52 (–6%)
ΔΣSUVmax_M3M6_ (%)	*p* = 0.49 (–14%)	*p* = 0.28 (–14%)	*p = 0.28 (−9%)*
ΔNOMV_M3M6_ (%)	*p* = 0.17 (–8%)	*p* = 0.09 (–8%)	*p* = 0.15 (–16%)
ΔTLOM_M3M6_ (%)	*p* = 0.17 (–10%)	*p* = 0.09 (–10%)	*p* = 0.15 (–16%)

*p = p-value (threshold used to dichotomize the 2 groups). In italic: better prognosis in the group with higher scintigraphic parameters or with worsening scintigraphy*.

* *statistically significant difference between the 2 groups. SUV, Standardized Uptake Value; NOMV, Neoplastic Osteoblastic Metabolic Volume; TLOM, Total Lesion Osteoblastic Metabolism*.

## Discussion

The role of bone scintigraphy in the therapeutic assessment of BmCRPC is fundamental. Indeed, bone metastasis are highly frequent, and, because they are mostly osteoblastic, they are not evaluable by the CT RECIST 1.1 criteria (6). Even if SPECT-CT improved the diagnostic performance compared to planar scintigraphy (8, 16), the therapeutic assessment according to the visual criteria proposed by the PCWG2 criteria has, to the best of our knowledge, never been validated. The aim of this study was to assess the feasibility of a therapeutic assessment of BmCRPC treated by abiraterone by using quantitative bone SPECT parameters and to compare their prognostic interest. However, the prognostic interest of these parameters has to be cautiously interpreted considering the small size of the population (19 patients).

We first showed the feasibility of quantitative therapeutic assessment of BmCRPC. We encountered technical issues in the first months of the process, resulting in a loss of data concerning some early patients. These issues were due to compatibility problems of the software with SPECT quantification, and were quickly solved, allowing then an easy quantitative therapeutic assessment. We think it could be performed in routine practice.

We found that the SUVmax of the highest uptake lesion at M0 seemed to be predictive of OS (*p* = 0.04). Pre-treatment SUVmax is a well-known FDG PET prognostic factor of other malignancies (17, 18). To the best of our knowledge, this study is the first one to report this prognostic SUV-based approach in bone SPECT in 19 patients with BmCRPC. Metabolic tumor volume has been reported as a better prognostic marker than SUV in several PET oncology series (17, 19). It was not the case in our study, maybe because of the small size of the population. NOMV and TLOM were not predictive of OS (*p* = 0.24 for each) or DSS (*p* = 0.41 for each) in our study. However, both low NOMV and TLOM were predictive of longer PFS (*p* = 0.02 and *p* = 0.04, respectively). This suggests that low tumor burden (oligo-metastatic BmCRPC) could be predictive of long-term response to abiraterone, which was already advocated by a previous study (20).

We used SUVmax and SUVpeak as bone SPECT-CT biomarkers by analogy with PERCIST (11) and EORTC 99 (12) criteria. Indeed, decrease of tumor SUVmax and/or SUVpeak on FDG PET after chemotherapy has been shown to be predictive of OS and PFS in many malignancies (11). New PET radiotracers have recently emerged in the assessment of prostate cancer (21). De Giorgi et al. (22) have shown that the decrease F-Choline uptake was predictive of longer PFS and OS in 43 patients with advanced prostate carcinoma treated by abiraterone (22). Assessment of response was based on the decrease of the SUVmax, based on EORTC 99 criteria. More recently, Uprimny et al. (23) have shown that primitive tumor SUVmax was correlated with PSA and Gleason score in PSMA PET-CT (23). These data show that tumor metabolism is a major prognosis factor in prostate cancer.

We performed this study on the assumption that osteoblastic metabolism reflected tumor activity. Indeed, this metabolism is increased in case of bone aggression. Sterilized bone metastasis show no osteoblastic metabolism because the bone is no longer inflamed by tumor cells. On the other side, the more tumor cells are viable, the more peritumoral bone matrix is active. The exception is flare-up phenomenon, corresponding to bone healing in the first weeks of treatment. We decided to take this phenomenon into account by assessing the therapeutic response between M3 and M6. Based on these physiological data, we assessed that the intensity of bone metabolism could be correlated with the tumoral burden.

Our results suggested that a TLOM increase of 20% between M0 and M6 could be a predictive factor of a shorter OS (*p* < 0.04). Among the 10 patients in whom TLOM could be measured at M0 and M6, 4 showed an increase of TLOM higher than 20% and 3 out of these 4 patients died during the follow-up. In the remaining 6 patients, only one died during the follow-up. Nevertheless, M0–M6 variation of TLOM was not significantly predictive of DSS (*p* = 0.24), due to a lack of events during this short period of follow-up. Finally M0–M6 NOMV and TLOM increases of at least 45 and 40%, respectively, seemed to be predictive of shorter PFS. Patients with increase of NOMV higher than 45% were considered as progressive because of PSA increase. There were considered as non-progressive according PCWG2 bone scan criteria. M0–M6 variations of NOMV or TLOM seem to be promising biomarkers of progressive disease.

Early worsening of the quantitative bone SPECT was of good prognosis. In effect, patients who showed a greater increase of the SUVpeak (more than 12.5%) of the highest uptake lesion had a longer OS (*p* = 0.03). Moreover, patients who showed an increase (higher than +4.2% in our population) of the SUVmax of the highest uptake lesions between M0 and M3 had a longer DSS (*p* = 0.01). This could be explained by the flare-up phenomenon. Indeed, since Tc99m-DPD is an indirect radiotracer of bone metastases, early new uptakes can reflect the healing of unseen lesions on M0. Increase of uptakes of previously seen lesions could also correspond to flare-up phenomenon. Even if the flare-up phenomenon had already been described, this study suggests that it could be of good prognosis in BmCRPC treated by abiraterone, even if there is no new lesion between M0 and M3.

Finally, there was a association between PSA variations between M0 and M6 and NOMV/TLOM variations. It was not the case for the SUVmax variations. This association suggests that the TLOM decrease between M0 and M6 could be predictive of longer OS, decrease of PSA being a well-known prognostic parameter (24). There was no association between baseline PSA and quantitative bone SPECT parameters.

Our study had some limitations. Firstly, only bone lesions were taken into account. This could explain some discrepancies between quantitative bone scan parameters and PSA variations that reflect the whole disease, including particularly lymph node involvement. Secondly, due to an absence of data in the literature, we arbitrary chose a SUV fixed value of 12 to delineate the NOMV. It appeared to be a good a compromise to delineate uptakes. Thirdly, SUV used in our study was normalized by the body weight. Nevertheless, a normalization by the skeletal volume could improve the quantification accuracy (25). Indeed, as Tc-99m DPD uptake is mainly contained into bone, body weight normalized SUV is higher in patients with high body mass index. Fourth, our population is relatively small and our preliminary conclusions have to be validated in further larger series. Finally, because of technical issues, quantification was not available for one of the two bed SPECT acquisitions in 4 patients at M0, and in 1 patient at M3. However, they all had many quantifiable bone malignant uptakes on the remaining images with the SUV based method. Only the NOMV and TLOM approaches were not applicable to these 5 cases in our statistical analysis.

## Conclusions

Our study shows the feasibility of using a quantitative therapeutic assessment of BmCRPC. Baseline SUVmax was predictive of OS. The early increase (between M0 and M3) of SUVpeak of the highest uptake lesion was predictive of longer DSS and OS, and could correspond to the healing of metastases (“flare-up” phenomenon). The decrease of TLOM between M0 and M6 seemed to be predictive of a longer OS. Quantitative bone SPECT-CT is a very promising approach to improve the accuracy of the therapeutic assessment of BmCRPC. Further studies using this tool in wider population are needed to generate new criteria of BmCRPC therapeutic response.

## Data Availability Statement

The datasets analyzed in this manuscript are not publicly available. Requests to access the datasets should be directed to DRCI Brest, dpo@chu-brest.fr.

## Ethics Statement

The studies involving human participants were reviewed and approved by University Hospital of Brest ethics committee (29BRC17.0189). The patients/participants provided their written informed consent to participate in this study. Written informed consent was obtained from the individual(s) for the publication of any potentially identifiable images or data included in this article.

## Author Contributions

RA, RL, and P-YS are the guarantors of the paper and designed the study. US, J-PM, VB, and FS ensured inclusion and follow-up of patients. RA, PR, SQ, OD, RL, and P-YS managed imaging procedures. RA, RL, and DB analyzed the data. RA and RL realized statistics. All authors contributed in drawing up the manuscript.

### Conflict of Interest

P-YS received a research grant from Siemens Molecular Imaging. The remaining authors declare that the research was conducted in the absence of any commercial or financial relationships that could be construed as a potential conflict of interest.
